# Biological Profile of Erucin: A New Promising Anticancer Agent from Cruciferous Vegetables

**DOI:** 10.3390/toxins2040593

**Published:** 2010-04-05

**Authors:** Antonietta Melchini, Maria H. Traka

**Affiliations:** 1 Foundation “Prof. Antonio Imbesi”, Pharmaco-Biological Department, School of Pharmacy, University of Messina/Villaggio Annunziata, 98168 Messina, Italy; Email: amelchini@pharma.unime.it; 2 Plant Natural Products and Health Programme, Institute of Food Research/Norwich Research Park, NR4 7UA Norwich, United Kingdom

**Keywords:** anticancer agents, isothiocyanates, erucin, rocket salads

## Abstract

Consumption of cruciferous vegetables has been associated with a reduced risk in the development of various types of cancer. This has been attributed to the bioactive hydrolysis products that are derived from these vegetables, namely isothiocyanates. Erucin is one such product derived from rocket salads, which is structurally related to sulforaphane, a well-studied broccoli-derived isothiocyanate. In this review, we present current knowledge on mechanisms of action of erucin in chemoprevention obtained from cell and animal models and relate it to other isothiocyanates. These mechanisms include modulation of phase I, II and III detoxification, regulation of cell growth by induction of apoptosis and cell cycle arrest, induction of ROS-mechanisms and regulation androgen receptor pathways.

## 1. Introduction

The recent report by the World Cancer Research Fund (WCRF) International together with the American Institute of Cancer Research (AICR) underlined that cancer is 30%–40% preventable over time by appropriate food and nutrition [1]. A significant part of the research on plant foods and cancer prevention suggests the potentially beneficial effect of a diet rich in cruciferous vegetables [2,3,4,5,6,7,8,9,10,11]. At present, the putative role of cruciferous vegetables on cancer chemoprevention is related to the bioactivity of the glucosinolate (GLS) hydrolysis products, namely isothiocyanates (ITCs), suggested to protect against the most common cancer types, such as lung, prostate, breast and colon cancers [12,13,14,15,16,17,18,19,20,21,22]. Among cruciferous vegetables, rocket salads are widely used in the Mediterranean diet and are well-studied as source of healthy phytochemicals [23,24]. The name “rocket” is commonly used to indicate different species belonging to the large family of Brassicaceae that are mainly represented by *Eruca sativa* Mill. (also known as salad rocket) and *Diplotaxis tenuifolia* L. (wild rocket). Both, largely distributed in the Mediterranean area, have gradually spread to other latitudes, and are used for their pungent flavor as new ingredients in green leafy salads [25]. In the last decade, salad species consumption has become increasingly important worldwide, encouraged from the positive link between eating fresh raw materials and absorption of health-promoting phytochemicals. It has been shown that the overall average bioavailability of ITCs is 61% and 10% for raw and cooked cruciferous vegetables, respectively [26]. In terms of antioxidant compounds, rocket salad species are a good source of vitamins, like vitamin C, carotenoids, and polyphenols, which play a very important role among natural antioxidants [27]. Moreover, they are characterized by high GLS content, such as other cruciferous vegetables, and their enzymatic degradation products are not only responsible for the typical sensory properties of these cruciferous salads, but also of their potentially beneficial effects on human health [28]. The 4-(methylthio) butyl isothiocyanate (erucin or ER) has been previously identified as a major component derived from rocket salad leaves [29,30], able to affect selectively cancer cell growth [31,32]. In this review, we attempt to provide an overview of the biological profile of this new cancer chemopreventive phytochemical, underlying its pharmacokinetic and pharmacodynamic properties. 

## 2. Pharmacokinetic and Bioavailability of ER

**Figure 1:**
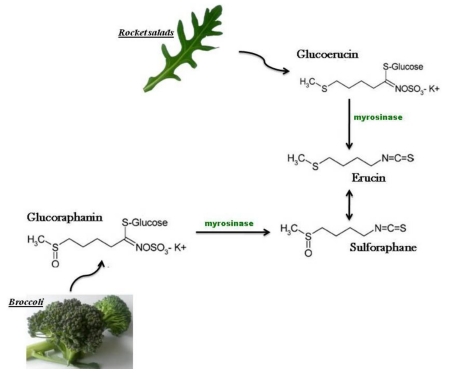
The 4-(methylthio)butyl isothiocyanate, erucin (ER), is a reduced analog of the 4-(methylsulfinyl)butyl isothiocyanate, sulforaphane (SF), and its formed both from enzymatic hydrolysis of glucoerucin, a glucosinolate found at high levels in rocket species (*Eruca sativa* Mill., *Diplotaxis tenuifolia* L.) and *in vivo* reduction of SF, derived from broccoli (*Brassica oleracea* L. ssp *italica*).

The isothiocyanate ER is obtained from enzymatic hydrolysis of glucoerucin, isolated for the first time in the 1970s from seeds of *Eruca sativa* Mill. and overall found at high levels in rocket salad species, but also through reduction *in vivo* of the isothiocyanate sulforaphane (SF), that structurally represents its oxidized analog, characteristic of broccoli (*Brassica oleracea* L. ssp. *italica*) [33,34,35] (Figure 1). 

The bioavailability of ER has not been studied to date. However, ER and SF are structurally related: both have an aliphatic side chain that could support a similar pharmacokinetic fate. SF, such as other ITCs, is initially subjected to enzymatic conjugation *in vivo* with the tripeptide γ-glutamylcysteineglycine (GSH) that is catalyzed by glutathione transferases (GSTs) [36]. The rate, however, of the enzymatic reaction of ITCs with GSTs was found to differ between structurally closely compounds. Among naturally occurring ITCs that differ only in the oxidation state of the sulfur atom that is inserted into the carbon chain, SF appears to be the poorest substrate for all four GST isoenzymes; in contrast, ER seems to be the best, thus its conjugation with GSH occurs faster compared to the reduced analog SF [37]. After conjugation with GSH, ITCs are principally metabolized by the mercapturic acid pathway. Mercapturic acids excreted in urine can be used to determine the bioavailability of ITCs, because their excretion in urine reflects the intake of GLSs, thus the corresponding uptake of ITCs, after cruciferous vegetables consumption [38]. It has been recently reported that ER and SF mercapturic acids are excreted in urine with high excretion levels after four hours following consumption of rocket salads (average bioavailability ≈ 94%) [26]. These data have underlined the same *in vivo* kinetics of these structurally related ITCs, showing similar absorption rate (ER k_a_= 2.5 h^−1^, SF k_a_= 2.0 h^−1^) and excretion rate (ER k_a_= 0.24 h^−1^, SF k_a_= 0.19 h^−1^) constants after rocket salad consumption. Interestingly, the excretion in urine of ER mercapturic acid has been also reported after consumption of cruciferous vegetables, such as broccoli and red and white cabbage that do not contain glucoerucin (<0.01 mmol/kg) [26]. After consumption of raw broccoli, not containing glucoerucin, both ER and SF mercapturic acids were found in urine samples after four hours with 29% and 50% excretion levels, respectively, where the level of excretion of ER mercapturic acid was expressed as % of the dose of glucoraphanin (the parent GLS of SF). These finding have shown for the first time the metabolic interconversion of SF to ER in humans. Previously, this metabolic reaction has been demonstrated in rats, where ER appears to be a major metabolic product of SF [35,39]. Approximately 12% of a single dose of SF, following intraperitoneal (ip) administration in rats, was eliminated in the urine (after 24 h) as *N*-acetylcysteine (NAC) conjugates of ER, and 67% of a single dose of ER was eliminated as conjugates of SF. These experimental data have assessed the reversibility of the oxidation-reduction biotransformation of the sulfur atom in ER and SF, showing that the oxidation of the sulfide in ER was a more favored metabolic reaction compare to the reduction of the sulfoxide in SF [35]. Moreover, the reduction *in vivo* of SF to ER has been demonstrated following oral administration of SF in rats, supporting the previous finding of intraperitoneal administration in rats [39]. Despite an insufficient direct knowledge of the metabolism of ER in humans, the *in vivo* interconversion of ER in SF and their structural similarity support the hypothesis about a similar metabolic fate. Thiol-conjugates of SF (thiol-SF) are the major metabolic products formed *via* mercapturic acid pathway in humans [38]. The conjugation of SF with thiols is a reversible reaction, and SF release by deconjugation reactions occurs in plasma and tissues under physiological conditions [40] and has been measured following broccoli consumption [41,42]. For this reason, the chemopreventive activity of both free SF and thiol-SF has been previously studied *in vitro* using different cancer cell lines, to reinforce the *in vivo* observation that SF and its metabolic products inhibit the progression of human cancer [43]. At present, the biological activity of ER has been investigated in various human cell lines, but there are no studies showing a similar *in vitro* bioactivity of its metabolic products.

## 3. Bioactivity of Rocket salad Species

Rocket species are well-known in traditional medicine for their therapeutic properties as an astringent, diuretic, digestive, emollient, tonic, depurative, laxative, rubefacient and stimulant [44,45,46,47]. It has been suggested that *Eruca sativa* seeds exert a beneficial antidiabetic effect in cases of chemically induced diabetes mellitus in rats by reducing oxidative stress [48]. *Eruca s*. extracts have also been shown to have a significant protective effect against HgCl_2_-induced nephrotoxicity in rats [49]. In both mentioned studies, the health-promoting activities of rockets plants have been partially related to their strong antioxidant properties [50,51,52]. Recently, anti-ulcer properties of rocket salads on experimentally-induced gastric secretion and ulceration in albino rats have been demonstrated [53]. In a study carried out by Lamy and colleagues there is evidence of the strong antigenotoxic effect of *Eruca s*. in benzo[a]pyrene exposed human hepatoma (HepG2) cells [54]. Recently, chemoprotective properties of rocket leaves on human colon cancer cells have been also investigated [55] (Table 1).

**Table 1:** Experimental evidence supporting health promoting activity of rocket salad species.

Rocket species	Health promoting activity	Experimental Model	Reference
*Eruca sativa* (seeds)	antidiabetic activity	rats	[48]
*Eruca sativa* (seeds)	protective effect against HgCl_2_-induced nephrotoxicity	rats	[49]
*Eruca sativa* (seeds, sprouts)	antioxidant activity	*in vitro* assays	[50]
*Eruca sativa* (leaves)			[51]
*Eruca sativa* (leaves)			[52]
*Eruca sativa* (leaves)	anti- ulcer activity	albino rats	[53]
*Eruca sativa*	antigenotoxic activity	human hepatocellular carcinoma HepG2 cell line	[77]
*Eruca sativa* (leaves)	chemopreventive activity	human colonic cancer HT-29 cell line	[55]
*Diplotaxis tenuifolia* (leaves)			

## 4. Cancer Chemoprevention and Treatment with ER: Evidence from Cell and Animal Research

ER has demonstrated promising anticancer effects in various *in vitro* and *in vivo* studies. Zhang and colleagues have shown for the first time the protective effects of ER against cancer through induction of detoxification enzymes in several mouse tissues [56]. These findings have been consequently confirmed in other rat and human tissues and in human cancer cells [57,58,59,60,61,62]. The effects of ER on growth inhibition, cell cycle regulation, and apoptosis induction in prostate, lung, liver and colon cancer systems have also been reported [31,54,62,63]. Cell cycle arrest, apoptosis and mitochondrial potential depolarization by ER was shown in human leukaemia cells and their multidrug resistance variants [64]. Moreover, ER can be considered a naturally occurring ITC able to affect selectively cancer cell growth, as shown in human leukemia cells. Fimognari and colleagues have provided evidence that ER is able to induce a strong antiproliferative effect on human leukemia cells, but not in non-transformed human peripheral T lymphocytes [32]. In a number of studies ER has been shown to share similar biological activity with SF [50,58,59,62,64], however SF seems to inhibit the growth of both transformed and non-transformed human peripheral T lymphocytes [65]. Although the phenyl ethyl isothiocyanate (PEITC) has exhibited selectivity for oncogenically transformed ovarian epithelial cells [66], ITC selectivity is an area of great interest that warrants further investigation.

## 5. Molecular Mechanisms of Cancer Chemoprevention by ER

**Figure 2:**
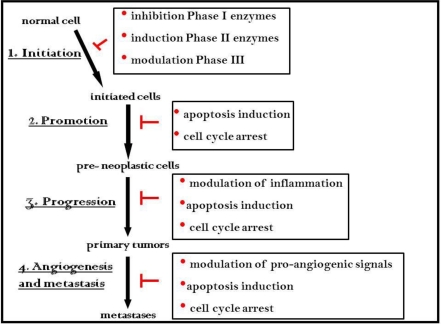
Molecular mechanisms of chemoprevention by ITCs, mainly determined through work on sulforaphane, an ITC related to erucin.

Our understanding of the mechanisms by which bioactive food components may prevent cancer is of primary importance for the translation of laboratory findings to clinical approaches. It is becoming increasingly clear that many dietary agents, such as ITCs, can retard or prevent the process of carcinogenesis by multiple mechanisms perturbing the three major steps, initiation, promotion and progression, as well as the later stages, angiogenesis and metastasis (Figure 2). 

The *in vivo* interconversion of ER to its structurally related analog SF and their structural similarity has suggested a similar biological activity. Since the early 1960s, when SF was discovered, its biological activity and molecular targets have been widely investigated *in vitro* and *in vivo* [36]. The growing body of cell and animal research has contributed to a comprehensive biological profile of SF by underlying several modes of action for its anticarcinogenic activity, such as enhancement of detoxification of human carcinogens through induction of phase II drug metabolizing enzymes, reduction of carcinogen activation through suppression of cytochrome P450-dependent monooxygenases, promotion of apoptosis in cancer cells, perturbation in cell cycle progression and inhibition of angiogenesis and metastasis [36]. At present, literature data suggest that ER may also exert its potential protective effects against human cancer through similar multiple mechanisms that will be summarized in the following subsections (Table 2).

**Table 2:** Chemopreventive effects of ER and its molecular targets *in vitro* and *in vivo* assays.

Biological activity	Molecular targets	Experimental model	Reference
Modulation of Phase I enzymes	CYP540 isoforms	human hepatocellular carcinoma HepG2 cell line	[77]
		*ex vivo* rat and human tissues	[57,58]
Induction of Phase II enzymes	quinone reductase (QR) glutatione transferase (GST)	mouse tissues	[56]
		human tissues	[61]
		human colonic cancer CACO-2 cells	[62]
		rat and human tissues	[59]
Up- regulation of Phase III detoxification system	multidrug resistance proteins (MRP- 1 and 2)	human colonic cancer CACO-2 cells	[61]
		human cancer cell lines (HepG2, CACO-2, A549)	[91]
Modulation of cell proliferation	tumour suppressor proteins (p53, p21)	human lung adenocarcinoma A549 cells	[31]
		human hepatocellular carcinoma HepG2 cells	[54]
	Cell cycle checkpoints	human colonic cancer CACO-2 cells	[61]
		human leukemia cells	[64]
			[32]
	pro- apoptotic signals	human colonic cancer CACO-2 cells	[61]
		human leukemia cells	[64]
			[32]
		human hepatocellular carcinoma HepG2 cells	[54]
	androgen receptor (AR)	human prostate cancer LNCaP cell line	[63]
	reactive oxygen species (ROS)	human acute myeloid leukaemia HL60 and erythroblastic chronic myelogenous leukemia K562 cell lines	[105]

To determine biological activity using in vitro and *in vivo* systems, it is essential to know the levels of ITCs achieved in systemic circulation following consumption of cruciferous vegetables. Although currently there is no evidence for ER and its metabolites in human plasma, there is evidence that SF and its metabolites circulate in the plasma of volunteers at a maximum concentration of 2.2 µM at 1.5 hours after consumption of one portion of broccoli, of which more than 40% was measured to be free SF [42]. This suggests that care should be exercised when extrapolating from cell studies where typical concentrations are several fold higher than those obtained through dietary consumption. However, it is likely that tissues of the upper gastrointestinal tract will be exposed to concentrations higher than other tissues that rely on systemic circulation. Yet, it is also conceivable that in those latter tissues ITCs could accumulate, thereby increasing concentrations above those found in systemic circulation.

### 5.1. Effects of ER on xenobiotic metabolism processes

The human metabolism is a major component of the physiological defense response to a large number of xenobiotics, including human carcinogens. Lipophilic compounds are converted to water soluble and readily excretable metabolites by enzymatic biotransformation reactions involving an initial introduction of functional groups (phase I) and the subsequent conjugation of such functional groups with endogenous polar molecules (phase II). The final excretion of xenobiotics and their metabolites is mediated by phase III transporters, which are subjected, as phase I and II enzymes, to regulatory mechanisms of both induction and inhibition. Several studies have identified the correlation between expression of various metabolizing enzymes with risk of different cancer types, highlighting the role of xenobiotic metabolic enzymes on carcinogenesis [67]. Many dietary compounds can directly activate or inhibit these enzymes perturbing xenobiotic metabolism [68]. 

Although SF has been shown to inhibit, directly or *via* a competitive mechanism, expression and activity of various phase I cytochrome P450 (CYP450) enzyme isoforms in rat and human tissues [69,70,71,72,73,74,75,76], phase I enzyme inhibition by ER has not been demonstrated to date. ER (5–20 μM) did not inhibit CYP1A1 protein expression in HepG2 cells after exposure to the human carcinogen benzo[a]pyrene (BaP 50 μM), although ER reduced the BaP-induced CYP1A1 activity in a dose-dependent manner reaching 25% inhibition at the highest concentration tested. Moreover, exposure of HepG2 cells with a concentration of 1 μM ER decreased the BaP-induced DNA migration by 50%. Interestingly, one of the ITC constituents of *Eruca sativa*, identified as erysolin, showed stronger activity compared to ER by inhibiting CYP1A1 activity in BaP-treated HepG2 cells by 50% at lower concentrations (5 μM) [77]. Despite the evidence from cell models, ER administered to rats at a dose of 3mg/kg day, corresponding approximately to human dietary intake, was able to only increase CYP1A1 in the lung and CYPB1B1 in both liver and lung tissue [57]. However, in the same study the correlation between the protective effects against chemically induced mutagenesis and the decrease of the formation of active mutagen intermediates by ER was demonstrated. Similarly, the bioactivation of a chemical carcinogen, 2-amino-3-methylimidazo-(4,5-f)quinoline (IQ), in ER pre-treated rats, was clearly decreased after ER pre-treatment, as previously shown following treatment with the structurally related ITC, SF [57,70]. 

These findings obtained in animal systems have been subsequently confirmed in human tissues. Hanlon and colleagues [57] studied the modulation of CYP450 enzymes expression and activity in human tissues after treatment of human liver slices with ER. CYP450-mediated dealkylations were not inhibited following ER treatment, with the exception of ethoxy-and methoxyresorufin slightly modulated at the highest concentration (50 µM). Although CYP1A2 and CYP1B1 apoprotein levels were markedly up-regulated by ER at a concentration of 10 µM in rat liver, a modest increase was observed in one of the human tissue samples tested. The observed inhibition of phase I enzymes by both ER and SF on rat and human tissues at higher concentrations may be related to the toxicity of high ITC concentrations rather than a real effect on phase I enzymes.

Despite the absence of an effect of ER in reducing phase I enzymes, phase II enzymes seem to be modulated similarly to SF by ER. Compared to SF, however, ER is less potent in inducing quinone reductase (QR) activity in murine hepatoma cells and both QR and glutathione transferase (GST) activities in mouse tissues [56]. This might be due to the difference in side chain length and oxidation status of sulphide sulphur between the ITC analogs. 

Interestingly, induction of phase II detoxification enzymes by ITCs reported in rat tissues exhibits organ selectivity. ER induced GSTs activity in the urinary bladder and in the forestomach, and QR activity in the urinary bladder and in the duodenum. In all other organs studied, such as liver, kidney, spleen, lung, heart, glandular stomach, jejunum, ileum, cecum, or colon plus rectum of rats, following intake of ER, or as well of SF, there was no effect on QR and GST activities. This observed organ selectivity could be explained by their pharmacokinetic properties. Since N-acetylcysteine conjugates of ITCs are excreted *via* the kidney, and deconjugated within the urinary bladder resulting in re-absorption of free ITCs in the bladder epithelium, the exposure of bladder epithelial cells to the ITCs could occur at higher concentration compared to other organs [78,79]. 

In a human colon carcinoma model, ER was shown to significantly induce phase II enzymes. Using undifferentiated human colonic Caco-2 cells, 20 µM ER was able to induce mRNA expression of QR (fold increase, ER = 11.1; SF = 3.3) and UDP-glucuronosyl transferase (UGT1A1) (fold increase, ER = 11.6; SF = 5.3), showing a stronger bioactivity than SF. It is already known that induction of phase II enzymes by ITCs is associated with the nuclear factor-E2-related factor 2 (Nrf2)-Kelch-like ECH-associated protein 1 (Keap1)-antioxidant response element (ARE) signaling pathway [36]. The cis-acting antioxidant response element (ARE 5’-(G/A)TGA(G/C)nnnGC(G/A)-3’) is a specific DNA-promoter-binding region, which is found in the 5’- flanking region of the phase II and antioxidant genes [80]. The ARE-driven gene transcription is regulated partially by nuclear factor Nrf2, which under normal conditions is sequestered in the cytoplasm by Keap1). In oxidative stress conditions or following cellular exposure to certain chemopreventive agents, Nrf2 is dissociated from Keap1 and translocated to the nucleus, where it binds to AREs and transactivates phase II detoxifying and antioxidant genes. The Nrf2-Keap1-ARE signaling pathway appears to be modulated by ER through different kinases, such as phosphatidylinositol 3-kinase (PI3K) and mitogen-activated protein kinases (MAPKs). Inhibitors of PI3K/Akt and Raf/MEK/ERK pathways decreased ER-induced phase II enzyme mRNA in undifferentiated CACO-2 cells [62]. 

In a model of lung cancer, both ER and SF were able to induce QR and GST expression and activities in rat lung tissue in a dose-dependent manner and with similar potency. QR protein levels were increased three-fold in rat lung slices following 24 hours incubations with both dietary ITCs, and similarly, but less markedly, GST protein levels were also elevated [58]. 

Recently, significant evidence of the inductive effects of ER, and its structurally related analog SF, on detoxifying enzymes in human and rat liver have been provided. ER and SF were shown to cause a statistically significant induction of QR and GST protein levels and activities in rat liver tissues up to 50 µM, but higher concentrations returned QR and GST to control levels [59]. SF appeared to be more potent than ER. Neither compound had any significant effect in human liver tissues, however, except for a modest increase in NAD(P)H: quinone oxidoreductase-1 (NQO1) protein levels and in both GSTα and GSTµ, but not in GSTπ protein levels, in only one of the two human samples. In particular, the induction of GST activities by the two dietary compounds was dependent from the substrates used, the 1-chloro-2,4-dinitrobenzene (CDNB) that is associated with a number of cytosolic transferases, the 7-chloro-4-nitrobenzo-2-oxa-1,3-diazole (CNBOD) and the 1,2-dichloro-4-nitrobenzene (DCNB), substrates for the α- and π-classes, respectively. ER was able to up-regulate GST activity only when CDNB and CNBOD, but not DCNB were used. In contrast, SF was effective when all three substrates were used. These findings suggested an isoform-specific induction of GSTs expression and activity by both ER and SF in liver from rat and human [59]. 

In addition to phase I and phase II modulation, several studies suggest that phase III efflux is also modulated by ITCs. Phase III detoxification system involves efflux pumps that actively eliminate toxic substances from inside cells. It has been established that membrane proteins, notably multidrug resistance (MDR), multidrug resistance protein (MRP), and breast cancer resistance protein (BCRP) of the ATP binding cassette (ABC) transporter family encoding efflux pumps, play important roles in the development of multidrug resistance [81,82,83,84]. Amino acid sequence analyses revealed that all these multidrug-resistance proteins contain multiple transmembrane domains (TMDs) and intracellularly localized ATP binding cassette (ABC) [85]. These multiple TMDs form a pore whereby animal cells use the intracellularly localized ABC to hydrolyze ATP to provide an energy source to eliminate cytotoxic compounds outward and reduce intracellular drug content to a sublethal level. The activities of these multidrug transporters can be up-regulated by many extracellular influences. Some of the up-regulation mechanisms are somewhat specific to particular drug transporters, but many are general and can affect many physiologic pathways [86,87,88]. Previously, SF was shown to increase MRP2 mRNA and protein expressions in primary human and rat hepatocytes coordinately with the induction of the detoxification enzymes QR and GST [89]. In human colon Caco-2 cells there was also an increase in MRP2 expression together with an increase in QR [90] and UGT1A1 [62]. In these cells ER (20 µM) was able to induce the mRNA of MRP2 by 6.7 fold, and with greater potency compared to SF (2.2. fold) [62]. ER also increased modestly MRP1 protein expression in hepatic carcinoma HepG2 cells at concentrations higher than 10µM, but the same effect was not observed in human cancer lung A549 and colon Caco-2 cells [91]. In contrast, MRP1 protein levels were increased by SF in a dose-dependent manner in all three cancer cell lines. Moreover, ER induced MRP2 in HepG2 and Caco-2 cells, but not in A549 cells, whereas SF was again effective in all three cell lines. 

### 5.2. Effects of ER on the physiological control of cell proliferation

**Figure 3:**
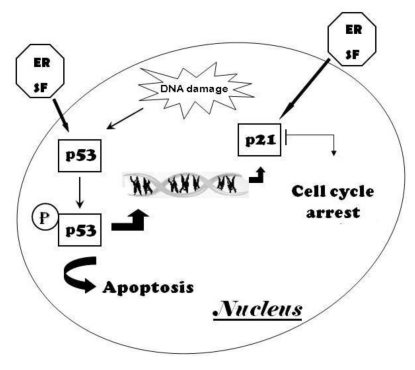
The tumor suppressor proteins p53 and p21 play a key role in the cellular response to DNA damage. Activation of p53 can lead to apoptosis in case of unrepaired DNA damage, or indirectly to the block of cell cycle progression by transactivating p21, a CDK inhibitor.

Several studies have showed the anti-proliferative activity of ER in different cultured cancer cells, providing mechanistic explanations that are in common to other aliphatic ITCs, such as SF, stressing the relationship between structure and biological activity of ITC analogs. Induction of cell cycle arrest and apoptosis seems to be an important molecular mechanism to explain the chemoprevention by ITCs [64,92,93,94,95,96,97]. The best understanding of how these phytochemicals impact on fundamental cellular processes, including the cell cycle, will come by understanding cancer biology first. The normal growth and replication of cells is carefully regulated by several types of genes and factors that control their expression. The tumor suppressor protein p53, also known as ‘‘guardian of genome”, plays an essential role in the control of cell proliferation. p53 is normally maintained at low levels in unstressed mammalian cells, but in response to cellular stress its accumulation in the nucleus of cells promotes its activation and stabilization by molecular modifications. The active p53 acts as regulator of the expression of a wide variety of genes involved in apoptosis and growth arrest [98] (Figure 3). Therefore, p53 can inhibit cell cycle by transactivating the Cyclin-Dependent Kinase (CDK) Inhibitor p21^WAF1/CIP1^ (p21). 

The p21 protein is an inhibitor of CDKs that regulates the cell cycle by inhibiting both the G1-to-S and G2-to-mitosis transitions [99]. Recently, our laboratory has demonstrated that ER up regulates in a dose-dependent manner the protein expression of p53 and p21 to inhibit the proliferation of human lung cancer A549 cells [31]. Compared to SF, ER showed lower potency in inhibiting proliferation of A549 cells (ER IC_50_ = 97.7 µM; SF IC_50_ = 82.0 µM), as well as in modulating p53 and p21 protein expression. Consistent with induction of apoptosis, we also demonstrated that poly (ADP-ribose) polymerase-1 (PARP-1) cleavage occurs after ER treatment in A549 cells, and similarly after SF treatment, as previously reported [100]. (PARP-1) is a protein involved in DNA repair in response to environmental stress. This protein acts by releasing apoptosis inducing factor (AIC) causing cell death, and its inactivation by proteolytic cleavage serves as marker of cells undergoing apoptosis. It was recently demonstrated that two ITCs, isolated in daykon sprouts (*Raphanus sativus* L.), also belonging to the family of Brassicaceae, are able to induce a strong cleavage of PARP-1 on human colon cancer cells [101,102]. SF and ER are saturated homologues of these ITCs, suggesting that PARP-1 cleavage is a mechanism for inducing apoptosis in a variety of cell tissues.

Previous studies have demonstrated the antiproliferative effects of ER in other cancer cell lines. The *in vitro* studies carried out by Fimognari and colleagues have provided evidence that ER is able to induce a strong antiproliferative effect on human leukemia cells, but not in non-transformed human peripheral T lymphocytes [32]. Moreover, cell cycle arrest in the G_2_/M phase and apoptosis induction by ER has been demonstrated in Caco-2 cells [62]. The percentage of Caco-2 cells in the G_2_/M phase was significantly increased following ER treatment compared to untreated cells that were mainly in the G_0_/G1 (56.4%) and in the S phase. The G2/M phase accumulation was accompanied by a corresponding decrease in G1 phase of the cell cycle. At higher ER concentration (50 µM), cell cycle distribution profiles reverted to those of control cells and a significant increase of sub-G1 apoptotic cells was observed. The percentage of apoptotic cells and necrotic cells after treatment with ER was also increased in a dose-dependent manner, showing the correlation between the reduction of Caco-2 cell survival and apoptosis induction [62]. 

Anti-proliferative effects of ER have also been observed in myeloid leukemia HL60 cells and its multidrug-resistant HL60/ADR and HL60/VCR sublines, with IC_50_ values of 1.9, 5.6 and 7.6 µM, respectively [64]. ER was able to induce a statistically significant increase of cells in the G_2_/M phase in both parental HL60 cells and multidrug-resistant HL60/ADR and HL60/VDR cell lines. Moreover, ER induced apoptosis and mitochondrial potential dissipation in these human cell lines, and was more effective than other aliphatic ITC analogs, such as iberin (IB) and SF [64]. 

Recently, Lamy and colleagues have reported the potential chemopreventive activity of ER in human hepatic cancer cells, and they have also correlated the significant bioactivity of this dietary compound with its *in vitro* biodegradation kinetics [54]. In HepG2 cells, ER is not detectable in the medium after 24 hours and only 25% is found after six hours. Similarly the degradation of ER in distilled water is time-dependent with a decrease of its starting concentration of 40%, 70% and 75% after one, six and 24 hours, respectively. Despite its degradation kinetics due to being volatile, ER was able to reduce HepG2 cell growth in a dose-dependent manner by inducing apoptosis already after 6 hours and cell cycle arrest at the G_2_/M phase, as well as increase p53 protein expression followed by an increase in p21 protein level. This suggests either that the short-term presence of ER is sufficient to elicit an effect, or alternatively that a proportion of ER binds to proteins in the medium thereby allowing for activation of downstream targets.

### 5.3. Effects of ER trough ROS-mediated mechanisms

Oxidative stress, resulting in excessive levels of free radicals and reactive oxygen species (ROS), is involved in the pathogenesis of chronic disease, and may be reduced by improving physiological antioxidant defenses through dietary interventions [103]. Rocket salad species have been considered a good dietary source of antioxidant compounds [29,30]. ER is characterized by direct antioxidant activity, because it is able to react with hydrogen peroxide and alkylhydroperoxides to form water and an alcohol [30,50], but also by indirect antioxidant capacity, being a potent inducer of cellular antioxidant systems, like the thioredoxin reductase 1 (TrxR1), as demonstrated in human breast cancer MCF-7 cells [60]. Recent work has correlated the pro-oxidant capacity of ITCs associated with ROS production with their chemopreventive properties [104]. It has been suggested that PEITC selectively killed transformed cells by increasing ROS production, because transformed cells have a higher level of ROS than non-transformed cells, and the further increase of ROS induced by PEITC treatment led to cell cycle arrest and apoptosis thereby preventing cancer cell proliferation [66]. ER also improved the cytotoxic effects of the arsenic trioxide (ATO), therapeutically used in the treatment of acute promyelocytic leukaemia (APL) through a ROS-dependent mechanism. In combination with ATO, ER may represent a promising therapeutic approach, by significantly increasing ATO-induced cytotoxicity in human leukaemia cells, acute myeloid leukaemia (HL-60) and erythroblastic chronic myelogenous leukemia (K562) cells, but not in promonocytic leukemia (U937) cells, through a ROS-dependent mechanism [105]. However, the ATO-induced cytotoxicity by ER was variable compared to erysolin and SF that significantly enhanced the growth inhibitory effects of ATO in all three leukemic cells lines, and for this reason ER was not subjected to further study [105].

### 5.4. Down-regulation of androgen receptor (AR) signaling pathway as novel mechanism of chemoprevention by ER

Androgen receptor (AR) is the most relevant signaling pathway in prostate cancer, and is presently considered a significant drug target, since its inhibition has demonstrated to be important for prostate cancer prevention and treatment [106,107,108]. Recent work suggests that increased consumption of broccoli and other cruciferous vegetables may have a significant benefit in reducing prostate cancer [3,4,5,109,110]. The protective effects of cruciferous vegetables consumption against prostate cancer appears to be partially related to the potential ability of ITCs to interact with the AR signaling pathway. PEITC perturbs AR signaling pathway by down regulating AR transcription through the inhibition of the AR gene promoter (Sp1) expression and inducing AR protein degradation in androgen-dependent (AD) and androgen-independent (AI) prostate cancer LNCaP cells [111]. These findings were supported by further observations that PEITC inhibited IL-6-induced AR-activation in androgen sensitive prostate cells [112]. Recently several *in vitro* studies also demonstrated that SF interacts with the AR pathway reducing prostate cancer survival [63,113]. Gibbs and colleagues have reported that SF treatment reduced protein levels of AR and its target genes, such as the prostate-specific antigen (PSA), by hyperacetylation of HSP90 in LNCaP cells [113]. SF treatment also decreased AR mRNA and protein levels, PSA secretion, and AR promoter activity in both LNCaP and C4-2 prostate cells [63]. A similar effects appears to be shared by naturally occurring thio analogs of SF, whereas sulfonyl analogs were inactive [63]. ER (0–10 µM) was also able to induce a statistically significant decrease in the expression of AR and PSA proteins in LNCaP cells [63]. 

## 6. Conclusions and Remarks

Pharmaceutical discovery of novel drugs for chronic diseases has turned many times to the plant world to identify promising bioactive phytochemicals. Epidemiological evidence and subsequent studies using cell and animal models have identified cruciferous vegetables as important sources of such phytochemicals that are emerging as novel potential anticancer agents. Although a lot of research has focused mainly on sulforaphane, derived from broccoli, and PEITC, derived from watercress, other ITCs such as ER are promising. In particular, there is evidence that ER is selective in its effects, inducing a strong antiproliferative effect on some human cancer cells, but not in non-transformed cells. This selectivity is an important characteristic that requires further investigation to identify amongst other things whether certain ITCs are selective on certain cancers. Although cell and animal models are essential tools to understand effects on and mechanisms of chemoprevention, it is important to direct research focus towards human intervention studies, using either isolated ITCs or whole foods, and discover whether the same mechanisms are also occurring in humans or whether food-derived compounds are working in a completely different way.
